# Pre-mRNA Splicing in Plants: *In Vivo* Functions of RNA-Binding Proteins Implicated in the Splicing Process

**DOI:** 10.3390/biom5031717

**Published:** 2015-07-24

**Authors:** Katja Meyer, Tino Koester, Dorothee Staiger

**Affiliations:** Department of Molecular Cell Physiology, Faculty of Biology, Bielefeld University, D-33615 Bielefeld, Germany; E-Mails: katja.meyer@uni-bielefeld.de (K.M.); tino.koester@uni-bielefeld.de (T.K.)

**Keywords:** Arabidopsis, splicing, RNA-binding protein

## Abstract

Alternative pre-messenger RNA splicing in higher plants emerges as an important layer of regulation upon exposure to exogenous and endogenous cues. Accordingly, mutants defective in RNA-binding proteins predicted to function in the splicing process show severe phenotypic alterations. Among those are developmental defects, impaired responses to pathogen threat or abiotic stress factors, and misregulation of the circadian timing system. A suite of splicing factors has been identified in the model plant *Arabidopsis thaliana*. Here we summarize recent insights on how defects in these splicing factors impair plant performance.

## 1. Introduction

**Figure 1:**
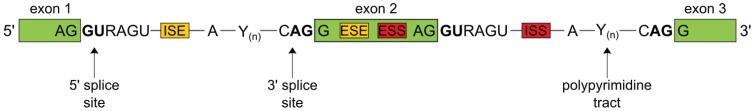
Splicing signals involved in pre-mRNA splicing. Introns are delineated by short consensus sequences, the 5' and 3' splice sites (R = Purine, A/G), the polypyrimidine tract (Y = Pyrimidine, C/T), and the branch point sequence. Intronic splicing silencers (ISS), intronic splicing enhancers (ISE), exonic splicing silencers (ESS), and exonic splicing enhancers (ESE) are additional sequence elements serving as docking sites for protein factors that promote or impair assembly of the spliceosome at particular splice sites and thus control the splicing process. The green boxes denote exons. Introns are represented by lines with the consensus sequences indicated (see text for details).

The realization of the genetic information encoded in eukaryotic genomes critically depends on the removal of intervening sequences (introns), the non-coding sequences, and the joining of the flanking exons through the splicing process [1,2]. The introns are delineated by short consensus sequences at the borders between the introns and exons, the splice sites (Figure 1). Splicing of the primary transcripts, the pre-messenger RNAs (pre-mRNAs), is accomplished by the spliceosome, a large complex consisting of several non-coding RNAs and a plethora of protein factors [3,4,5,6]. The process of pre-mRNA splicing is best understood in mammals and yeast, mainly through biochemical purification of spliceosomal components and functional tests using *in vitro* splicing assays.

### 1.1. The Splicing Process

The spliceosome assembles at each intron in a precise order, briefly summarized here [4]. The spliceosome comprises small nuclear ribonucleoprotein complexes (snRNPs) which are composed of small nuclear RNAs associated with distinct sets of proteins. The U rich small nuclear RNAs (U snRNAs) are short non-coding, non-polyadenylated transcripts. The Sm class of snRNAs, U1, U2, U4, and U5, associates with Sm proteins, named after the patient Smith with an autoimmune disease who developed antibodies against these proteins. The Like-Sm U6 snRNA associates with the related Sm-like proteins Like Sm 2 (Lsm2) to Lsm8 to form the U6 snRNP [7].

**Figure 2:**
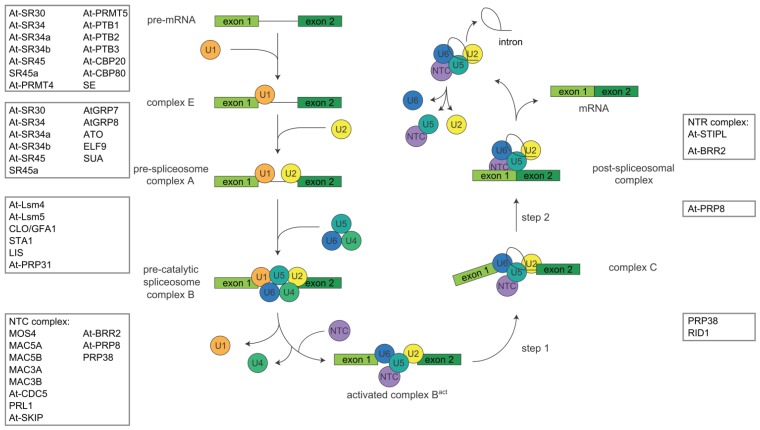
The spliceosomal cycle. The splicing process is initiated by the interaction of the U1 snRNP with the 5' splice site through base pairing of the U1 snRNA to complementary sequences. The U2AF subunit U2AF^35^ binds to the intron/exon border and the subunit U2AF^65^ binds to the polypyrimidine tract upstream of the intron/exon border, leading to the complex E. Subsequently, the U2 snRNP binds to the branch point, defining complex A. The U4/U6.U5 snRNP is then docked onto the U2 snRNP, leading to the precatalytic complex B. After major rearrangements and the release of the U1 and U4 snRNPs, the activated complex B^act^ is generated. In the first step of the splicing reaction, the pre-mRNA is cleaved at the 5' splice site and lariat formation of the intron sequence takes place by ligating its 5' end to the branch point adenosine. This leads to the formation of complex C that then catalyzes the second step of the splicing reaction, the cleavage at the 3' splice site, the ligation of the exons, and the release of the spliced mRNA. The lariat is degraded and the snRNPs are recycled. The proteins with a demonstrated role in pre-mRNA splicing in Arabidopsis are shown where in the spliceosomal cycle they are proposed to act, based on the homology to their human counterparts. The boxes denote exons; the thin line denotes the intron. NTC, Nineteen Complex.

The splicing process is initiated by the interaction of the U1 snRNP with the 5' splice site through base pairing of the U1 snRNA to complementary sequences in the pre-mRNAs (Figure 2). The two subunits of U2 auxillary factor (U2AF) interact with the 3' splice site. The 35 kDa subunit U2AF^35^ binds to the intron/exon border and the 65 kDa subunit U2AF^65^ binds to the polypyrimidine tract upstream of the intron/exon border, leading to the so-called complex E. Subsequently, the U2 snRNP binds to the branch point via base pairing of U2 snRNA, defining complex A. An assembly of the U4, U5, and U6 snRNPs (the so-called U4/U6.U5 snRNP) is then docked onto the U2 snRNP, leading to the precatalytic complex B that comprises all five snRNPs. After major rearrangements and the release of the U1 and U4 snRNPs, the activated complex B^act^ is formed. In the first step of the splicing reaction, the pre-mRNA is cleaved at the 5' splice site and lariat formation of the intron sequence takes place by ligating its 5' end to the branch point adenosine. This leads to the formation of complex C that then catalyzes the second step of the splicing reaction, the cleavage at the 3' splice site, the ligation of the exons, and the release of the spliced mRNA. The lariat is degraded and the snRNPs are recycled. Throughout the catalytic cycle, ATP-dependent RNA helicases are involved in the remodeling of the spliceosome, e.g., through reconfiguring RNA-RNA interactions [8].

### 1.2. Alternative Splicing

Not every splice site of a pre-mRNA is used in each instance the gene is transcribed. This conditional splicing is known as alternative splicing [9,10]. It enables the cell to obtain different mRNA isoforms from the same gene. By these means, the coding capacity of the genome is greatly enhanced. In humans, for example, alternative splicing expands the proteome five times compared to the number of protein-coding genes [11]. Alternative splicing events include exon skipping (ES), alternative 5' or 3' splice site selection, retention of introns (IR), or combinations of these events. Depending on the exclusion or inclusion of exons, protein variants with different domain composition arise from the mRNA isoforms. The corresponding protein variants may have distinct functions or localize to different subcellular compartments. Furthermore, alternative splicing can alter the open reading frame (ORF) and lead to mRNA variants with premature termination codons (PTCs). Such PTC-containing transcripts can be eliminated via the nonsense-mediated decay (NMD) pathway, a specialized RNA decay pathway that removes PTC-containing mRNAs that upon translation may give rise to aberrant proteins [12]. In effect, alternative splicing-NMD can thus also lead to altered transcript levels.

### 1.3. Regulation of Splicing

The use of the splice sites, *i.e.*, the assembly of the spliceosome at defined splice sites, is governed by *cis* regulatory elements that can be located both in the introns and the exons. These elements function by recruiting additional RNA-binding proteins during the assembly and the catalytic cycle of the spliceosome. These factors either promote or inhibit the use of splice sites. Accordingly, the regulatory sequence motifs are termed exonic splicing enhancers, exonic splicing silencers, intronic splicing enhancers, and intronic splicing silencers (Figure 1). The *trans*-acting proteins belong mainly to two broad categories. The SR (Serine Arginine rich) proteins consist of one or two RNA recognition motifs (RRMs) that contact the RNA targets and a domain enriched in serine and arginine residues involved in protein-protein interaction [13]. The hnRNPs (heterogeneous nuclear ribonucleoparticle proteins) are a class of RNA-binding proteins that associate with heterogeneous nuclear RNA and mRNA in the cytoplasm and participate in multiple steps of pre-mRNA processing in addition to their role in splicing [14]. How the splicing regulatory elements impact the choice of splice sites may vary depending on the sequence context in their surroundings. For example, SR proteins commonly repress splicing when they are bound by intronic sequences and activate splicing when they are bound by exonic sequences [15]. Mammalian hnRNPA1 inhibits splicing when recruited to introns or to exons [15]. The hnRNP-like proteins of the neuro-oncological ventral antigen (NOVA) family recognize YCAY (Y = pyrimidine, C or U) motifs that can act as exonic splicing silencers or serve as intronic splicing silencers when located in the intron preceeding an alternatively spliced exon or as an intronic splicing silencer when located in the intron following an alternatively spliced exon [16].

The importance of correct splicing patterns for the function of the cell is underscored by the observation that in humans, mis-regulation of splicing is a common feature of many diseases [17,18,19]. These disorders can be caused by mutations that disrupt the splicing of specific genes or by a general loss of spliceosomal function, affecting entire networks of downstream genes.

### 1.4. Pre-mRNA Splicing in Plants

Posttranscriptional control of gene expression by regulated pre-mRNA splicing has recently moved in the focus of plant molecular biology, as alternative splicing has been found to reshape the transcriptome in particular in response to biotic and abiotic stress [20,21,22]. Accordingly, aberrant pre-mRNA splicing leads to severe phenotypes including impaired pathogen defense and abnormal developmental programs [23,24,25,26,27]. Currently, it is estimated that 61% of all multi-exon genes undergo alternative splicing in the model plant *Arabidopsis thaliana*, whereas in mammals alternative splicing has been found for more than 90% of intron containing genes [28].

Knowledge on pre-mRNA splicing in higher plants is still limited. While the basic principles are conserved there appear to also be differences in detecting introns. A mammalian intron was not efficiently spliced in plants, but some plant introns were correctly spliced in HeLa cell extracts [29,30,31]. One of the differences may lie in the vastly differing lengths of introns from animals that are about 5 kb on average and of plant introns with an average length of 160 bp [32].

In Arabidopsis, genes for counterparts of most spliceosomal proteins have been identified through homology searches. Soon after the Arabidopsis genome was available, 395 genes for factors predicted to function in the splicing process were identified [33]. Later, Koncz and co-workers systematically mined the TAIR10 (The Arabidopsis Information Resource 10) version of the Arabidopsis genome for homologs of components of biochemically purified spliceosomal complexes in humans, Drosophila, or yeast that were identified via mass spectrometry [34]. This search yielded another hundred candidate splicing factors. In many cases, several paralogs of mammalian and yeast spliceosomal components have been found in Arabidopsis, pointing to some redundancy within these families [33,34]. Indeed, numerous mutants have been identified in Arabidopsis with defects in predicted counterparts of mammalian and yeast splicing factors while such mutants would not be viable in the absence of a compensatory gene.

Here, we summarize recent advances in understanding proteins involved in pre-mRNA splicing and alternative splicing in the reference plant *Arabidopsis thaliana*. We will not provide a complete inventory of splicing factors. Rather, we mainly focus on proteins with a demonstrated *in vivo* function in pre-mRNA splicing through mutant analysis or transgenic approaches and summarize the phenotypes generated by misexpression of the splicing factors. *Arabidopsis thaliana* proteins named according to their mammalian counterparts are designated with “At-” for clarity. In keeping with the convention in the plant community, Arabidopsis proteins are given in uppercase letters, the corresponding transcripts are given in uppercase and italics, and mutants are given in lowercase and italics.

## 2. Proteins Involved in Pre-mRNA Splicing in *Arabidopsis thaliana*

### 2.1. SR Proteins

The first proteins shown to regulate pre-mRNA splicing in plants were members of the SR protein family [35]. Originally, SR proteins in plants were identified through their cross-reaction with the mAB104 antibody directed against a phosphorylated serine in the RS domain in mammalian SR proteins [36,37]. Of note is the high number of SR proteins in plants. *Arabidopsis thaliana* has 18 SR proteins that represent six different gene families [35,38,39,40,41]. Three of them comprise orthologs of metazoan SR proteins. The family of ASF/SF2 orthologs comprises At-SR30, At-SR34, At-SR34a, and At-SR34b. The family of 9G8 orthologs comprises At-RSZ21, At-RSZ22, and At-RSZ22a that feature one zinc knuckle. In addition, there is one ortholog of SC35. The other three SR families are plant-specific. The RS2Z family comprises At-RS2Z32 and At-RS2Z33 with two zinc knuckles between the RRM and the RS domain and a C-terminal serine and proline-rich extension [42]. The RS family comprises At-RS31, At-RS31a, At-RS40, and At-RS41. The SCL family harbors At-SCL28, At-SCL30, At-SCL30a, and At-SCL33.

Ectopic expression of the SR proteins At-SR30 and At-RS2Z33 in transgenic plants produces a range of morphological and physiological phenotypes and impacts alternative splicing of a suite of other transcripts [43,44]. Recently, alternative splicing of At-SR30 and At-RS31 has been shown to be regulated by retrograde signaling from the chloroplast to the nucleus, a process that synchronizes gene expression in the nucleus with the needs of the chloroplast compartment [45]. Mutants deficient in At-SR34b are sensitive to Cadmium (Cd) due an increased uptake of toxic Cd^2+^ ions into the root [46]. In *sr34b*, splicing and stability of the mRNA-encoding IRON REGULATED TRANSPORTER 1 (*IRT1*), a transporter of Cd^2+^ ions located in the plasma membrane of root epidermal cells, are altered, suggesting that altered posttranscriptional regulation of *IRT1* in *sr34b* is linked to the Cd sensitivity phenotype.

Several SR proteins undergo auto-regulation through alternative splicing to PTC-containing isoforms [44,47,48,49]. About half of the PTC-containing isoforms are degraded via NMD and thus lead to alterations in transcript abundance [50,51]. Several of these alternative splicing events in SR genes are evolutionary conserved, underscoring their physiological relevance [41,52]. Recently, regulatory elements within an intron of *At-SCL33* have been identified that mediate auto-regulation of *At-SCL33* alternative splicing [53]. At-SCL33 binds to a conserved sequence motif in this intron with four GAAG motifs that are necessary for correct splicing. The identification of binding sites for the SR protein will help to identify additional downstream targets of At-SCL33.

In addition to the canonical SR proteins, Arabidopsis harbors the SR-like protein SR45 with two RS domains, one of each side of the RRM, that shows homology to RNA binding protein S1, serine-rich domain (RNPS1) of the mammalian exon junction complex, a protein complex loaded onto mRNAs upon splicing [54]. The *sr45* mutant exhibits developmental abnormalities, including narrow leaves, an altered number of petals and stamens in the flowers, and short roots [55]. The use of alternative 3' splice sites leads to two alternative splice isoforms which differ by eight amino acids. The short splice variant SR45.2 specifically complements the defect of the *sr45* mutant in root growth [56]. SR45.1 harbouring the T^218^SPQRKTG peptide specifically complements the defect of the *sr45* mutant in petal development [56]. The mutation of T^218^ to alanine abolished the complementation whereas the mutation of T^218^ to aspartic or glutamic acid, which mimics the charge of a phosphate group, still allowed rescue of the mutant [57]. This has been attributed to phosphorylation of T^218^. Additionally, the *sr45* mutant displays hypersensitivity to glucose and the phytohormone abscisic acid (ABA), which is complemented by both splice isoforms [58]. SR45 has recently also been implicated in splicing of the circadian clock gene *CCA1* (*CIRCADIAN CLOCK ASSOCIATED 1*). Through alternative splicing at its fourth intron, the *CCA1* transcript can give rise to transcript isoforms retaining intron 4 that would generate truncated protein isoforms due to PTCs. In the *sr45* mutant, less of the intron-retained forms are detected compared to wild-type plants. Because recombinant SR45 protein is able to bind to the retained *CCA1* intron *in vitro*, it may contribute to this alternative splicing event [59]. It is not yet known whether the *sr45* mutant exhibits a defect in circadian clock function. Furthermore, SR45 regulates alternative splicing of several *SR* transcripts, indicating extensive hierarchical regulation among those splicing regulators [55,60]. In the At-SR30 pre-mRNA, SR45 binds to the 5' end of intron 10, whose splicing is altered in the *sr45* mutant. SR45 interacts with the U1 snRNP protein U1-70K involved in the recognition of 5' splice sites and with U2AF^35b^ involved in the recognition of 3' splice sites, suggesting that SR45 helps to recruit U1snRNP to the 5' splice site and At-U2AF to the 3' splice site [60].

A hallmark of SR function is regulation by posttranslational modification. A large proportion of the SR proteins can undergo phosphorylation [61,62]. In particular, a LAMMER protein kinase, named for a conserved EHLAMMERILG motif in their catalytic subdomain, can phosphorylate SR proteins, and overexpression of the LAMMER kinase leads to aberrant splicing of At-SR30 and At-SR34 as well as other transcripts [63].

### 2.2. hnRNPs

The second type of splicing regulators described in plants corresponds to the hnRNPs [64].

#### 2.2.1. Polypyrimidine Tract Binding Proteins

In Arabidopsis, three homologs of the polypyrimidine tract binding proteins (PTBs) that interact with the pyrimidine rich motifs at the 3' splice sites have been identified. In mammals, PTBs either activate or repress the usage of specific splice sites in the vicinity of their binding sites. PTBs can also act in a combinatorial manner with other hnRNP proteins or SR proteins [65].

Mammalian PTB negatively auto-regulates by alternative splicing where skipping of the first exon produces an NMD substrate [66,67]. Arabidopsis At-PTB1 and At-PTB2 proteins similarly show negative auto-regulation by alternative splicing of their own pre-mRNAs where inclusion of a PTC-containing cassette exon creates an NMD substrate [68]. Furthermore, At-PTB1 and At-PTB2 reciprocally cross-regulate through the same mechanism. In transgenic Arabidopsis, overexpression or knock-down of At-PTB1 or At-PTB2 alters the splicing pattern of a plethora of transcripts [69,70]. Using a transient expression system it was shown that At-PTB1 promoted skipping of an alternative exon in a splice reporter construct whereas At-U2AF^65^ promoted its exclusion, indicating that At-PTB1 and At-U2AF^65^ can act antagonistically, similar to what is observed in mammals [70].

A physiological role for At-PTBs in seed germination was found through the control of *PHYTOCHROME INTERACTION FACTOR 6* (*PIF6*) splicing. A *PIF6* alternative splice variant is predicted to encode a short protein variant lacking the DNA binding domain due to exon skipping [71]. Down-regulation of AtPTB1 or AtPTB2 increased the exon skipping, and the shift to the short protein variant accelerated seed germination in the presence of the phytohormone abscisic acid (ABA) that retards germination. Up-regulation of At-PTB1 or At-PTB2 in turn reduced exon skipping, favoring the full length PIF6 protein, and delayed seed germination in the presence of ABA.

The more distantly related At-PTB3 did not exhibit a significant impact on alternative splicing [69].

#### 2.2.2. Glycine-Rich RNA Binding Proteins

Higher plants harbor a gene family encoding small RNA-binding proteins with an N-terminal RRM and a glycine stretch at the C-terminus that are simplified versions of mammalian hnRNPs [72,73]. These glycine-rich RNA-binding proteins are involved in responses to diverse environmental stresses. Two of the family members, AtGRP7 and AtGRP8, have a role in alternative splicing. These proteins are controlled by the circadian clock [74]. A reverse genetic approach has shown that AtGRP7 plays a regulatory role in the circadian timing system [75]. AtGRP7 negatively auto-regulates through binding to its own mRNA, leading to the usage of an alternative cryptic splice site within the intron that generates an alternative transcript isoform subject to NMD [76]. AtGRP8 uses the same negative feedback loop to control its own expression. Additionally, AtGRP7 and AtGRP8 are able to reciprocally cross-regulate each other’s expression [77]. Thus, they are the first example of a posttranscriptional feedback loop in the circadian timing system of any organism.

When transgenic plants with elevated or reduced levels of AtGRP7 were investigated with an alternative splicing panel based on Reverse Transcription–PCR [78], significant changes of alternatively spliced isoforms were found for 20% of the analyzed splicing events [79]. In particular, altered AtGRP7 levels lead to changes in the choice of alternative 5' splice sites. Several of the identified transcripts are bound *in vivo* by AtGRP7 but not by a mutant version with the conserved arginine 49 in the RRM that is important for RNA binding exchanged for glutamine [79,80]. This indicates that AtGRP7 impacts alternative splicing of these transcripts via direct interaction. Several of these alternative splicing events are also influenced by the paralog AtGRP8, indicating that both proteins act on a shared set of downstream targets. Notably, AtGRP7 also boosts plant defense reactions against pathogenic *Pseudomonas syringae* bacteria. It binds to several defense-related transcripts *in vivo* [81,82]. Upon infection, pathogenic *Pseudomonas syringae* bacteria inject type III effector protein HopU1, an ADP riboslyase, into plant cells as part of their virulence strategy [83]. HopU1 ADP ribosylates the R^49^ in the RNA recognition motif of AtGRP7 to disable RNA binding and subvert immune responses [84].

### 2.3. Spliceosomal Proteins

Apart from the involvement of SR proteins and hnRNPs in the regulation of splicing, mutants deficient in spliceosome-associated proteins have been found to exhibit splicing defects in Arabidopsis.

#### 2.3.1. General snRNP Proteins

The snRNPs of the spliceosome contain general proteins in addition to proteins specific for the respective snRNP. *SmD3b* mutants defective in the general snRNP core protein At-SmD3b display a range of physiological phenotypes including impaired root growth, an altered number of floral organs, and late flowering [85]. These phenotypes correlated with altered splicing patterns of several pre-mRNAs.

Mutants defective in the U6 snRNP core protein At-Lsm4 also show splicing defects [86]. In particular, several genes of the circadian clock are aberrantly spliced. Accordingly, the *lsm4* mutant shows a long period of circadian transcript oscillations [87]. The *lsm5* mutant shows a global defect in pre-mRNA splicing [88,89]. A reduced level of U6 snRNA in *lsm5* suggests that At-Lsm5 contributes to U6 stability [88]. In particular, At-Lsm5 affects the splicing of transcripts associated with salinity stress, a major threat to plants. Accordingly, constitutive overexpression of At-Lsm5 improves salt tolerance in transgenic plants. This correlates with an increased splicing accuracy and efficiency for stress-responsive genes [89].

Arginine methylation is an important posttranslational modification of proteins catalyzed by protein arginine methyltransferases. While many cellular proteins including histones can be modified by these enzymes, arginine methylation is frequently observed in RNA-binding proteins with glycine-arginine-rich motifs. Protein arginine methyltransferase 5 methylates the spliceosomal U snRNP proteins B, B', D1, and D3. This modification is crucial for the assembly of the snRNP complexes [90,91]. In Arabidopsis, *prmt5* mutant plants show a range of defects including late flowering. The transition from vegetative to reproductive growth is an important developmental decision in the life of the plant and accordingly is exquisitely controlled. In Arabidopsis, flowering is repressed by the key floral repressor FLOWERING LOCUS C (FLC) until the plant encounters favorable conditions. Late flowering of *prmt5* mutants correlates with elevated *FLC* levels. Furthermore, the *prmt5* mutant shows reduced inhibition of hypocotyl elongation in response to light and impaired root growth in the presence of high levels of NaCl, pointing to reduced salt stress tolerance [92,93]. The *prmt5* mutant has also a defect in circadian timekeeping, showing a longer period of leaf movement and gene expression rhythms [94,95]. At-PRMT5 affects splicing of the core clock gene *PSEUDORESPONSE REGULATOR 9* (*PRR9*), suggesting that the circadian defect in *prmt5* can be partly attributed to changes in *PRR9* splice patterns [94,96]. Moreover, At-PRMT5 deficiency causes global splicing defects. About 20% of investigated alternative splicing events were altered in the *prmt5* mutant plants. Moreover, constitutive splicing was affected, although to a lesser extent than alternative splicing [93]. In *prmt5*, methylation of the U snRNP At-SmD1, At-SmD3, and At-LSm4 proteins is disturbed, which may be responsible for the observed splicing defects [96].

Recently, Arabidopsis At-PRMT4, a representative of type I protein arginine methyltransferases that generates monomethylated and asymmetrically dimethylated arginine residues, was analyzed for a role in splicing control. This was prompted by the observation that among substrates for human PRMT4, also known as CARM1, are several splicing factors including the U1C protein, a component of the U1 snRNP, SAP49 and CA150, which are associated with the U2 snRNP, as well as the spliceosomal protein SmB. Moreover, PRMT4/CARM1 regulates splicing by promoting exon skipping [97]. Single mutations in either At-PRMT4a or At-PRMT4b did not cause major developmental defects, pointing to the redundancy of the two proteins [98]. Double mutants deficient in both At-PRMT4a and At-PRMT4b show strikingly similar phenotypes as *prmt5* mutants, including late flowering, reduced sensitivity to light, and reduced salt stress tolerance [93]. However, they are not affected in circadian timekeeping. Transcript profiling of the *prmt4a prmt4b* mutant revealed for the first time a global role for At-PRMT4 in the control of alternative splicing in any organism and also uncovered a function in constitutive splicing. Both At-PRMT4 and At-PRMT5 appear to act on introns with weak 5' splice sites that deviate from the consensus sequence. Thus, At-PRMT4 and At-PRMT5 function may be required to stabilize the interaction of the weak splice sites with U1 snRNA [93]. A genome-wide survey will be required to test whether the mammalian counterparts may have a similar bias towards weak 5' splice sites.

#### 2.3.2. U snRNP Specific Proteins

In addition to the general spliceosomal proteins, the function of several U snRNP-specific proteins has been characterized in Arabidopsis. STABILIZED 1 (STA1) is a homolog of the human U5 snRNP-associated 102-kDa protein, also known as PRPF6 (Precursor RNA Processing factor 6) [99]. STA1 affects splicing of transcripts that are induced by low temperature exposure of plants including the *COR15A* (*COLD REGULATED 15A*) transcript. *COR15A* encodes a polypeptide located in the chloroplast that protects chloroplast membranes against damage at low temperatures [100]. Because the *sta1* mutant is cold-sensitive, STA1 appears to be required for correct splicing during the cold stress response.

The spliceosomal protein PRPF31 regulates the formation of the U4/U6.U5 snRNP. While *prp31* null mutants in the Arabidopsis homolog of PRPF31 are embryonic lethal, mutants with a reduced level of At-PRP31 also show an impaired tolerance to low temperature [101]. At-PRP31 is required for splicing of cold-induced genes, especially under cold stress. Expression of *STA1* and *At*-*BRR2*, a homolog of the U5 snRNP-specific RNA helicase that acts at specific steps of the splicing cycle to catalyze RNA-RNA rearrangements and spliceosome remodeling, is up-regulated in the *prp31* mutant. This suggests a concerted action of these splicing factors in the low temperature response.

A genetic screen for Arabidopsis mutants with defects in the development of female gametes, based on aberrant expression of an egg cell specific reporter gene, yielded mutants defective in several snRNP proteins [102]. While homozygous mutants are lethal, the heterozygous mutants show severe defects during development of the female gametes. In the female gametophyte, two gametic cells, the egg cell and central cell, as well as several nongametic accessory cells, the two synergid cells neighboring the egg cell and three antipodal cells, develop. In *lachesis* (*lis*) mutants, supernumerary egg cells are observed due to misspecification of the synergids, the central cell and the antipodal cells that also express features of the central cell, *i.e.*, they aberrantly activate a gametic cell fate in a non-gametic cell [102]. LIS codes for a homolog of the U4/U6-specific PRPF4. *LIS* is up-regulated in gametic cells and down-regulated in the accessory cells. Mutants defective in CLOTHO (CLO)/GAMETOPHYTIC FACTOR 1 (GFA1) show diminished female gametophyte development and arrest of early embryo development [103,104]. GFA1 encodes a homolog of the U5–116kD protein and physically interacts with homologs of two other components of the U5 snRNP, the U5-200kD protein At-BRR2, and the U5-220kD protein At-PRP8. In *atropos* (*ato*) mutants with a defect in the homolog of SF3A3, a component of the U2 snRNP, the fate of the egg cell and central cell is also compromised [104]. Collectively, the phenotypes of these mutants indicate that correct splicing is essential for cell fate determination in the female gametophyte. Surprisingly, mutations in factors crucial for the splicing process have severe consequences only in a very limited developmental window, pointing to novel mechanisms of spliceosome function yet to be described.

Mutants in several spliceosomal proteins show alterations in the timing of the transition to flowering through misexpression of the floral repressor *FLC*. Antisense transcripts originating from a promoter downstream of the *FLC* gene play a crucial role in down-regulation of *FLC* expression through chromatin modification. These *COOLAIR* antisense transcripts themselves undergo alternative splicing and alternative polyadenylation either at a promoter proximal poly(A) site or a distal poly(A) site opposite to the *FLC* transcription start site. The use of the promoter proximal poly(A) site in the *COOLAIR* antisense transcript correlates with increased histone H3 lysine 4 demethylation and reduced expression of the *FLC* sense transcript [105].

A crucial role in *FLC* antisense transcript regulation has been found for the homolog of yeast PRP8, the largest and most highly conserved protein of the spliceosome [106,107]. PRP8 is necessary for several rearrangements occurring throughout the spliceosomal cycle.

While a complete loss-of-function mutant Arabidopsis At-PRP8 is lethal, a hypomorphic mutation reduces splicing of the *COOLAIR* introns and usage of the proximal poly(A) site [108]. This leads to increased histone methylation in the *FLC* gene and concomitant up-regulation of *FLC* transcription. Accordingly, the transition to flowering is delayed in *prp8*.

Arabidopsis harbors two homologs of a tetratricopeptide repeat protein with similarity to the yeast U1 snRNP-specific pre-mRNA processing protein PRP39. The *prp39a* mutant flowers later than wild-type plants and shows an elevated *FLC* level [109]. Whether splicing is affected in the *prp39a* mutant has not yet been investigated.

A key component promoting the transition to flowering in response to increasing day length in spring and summer is SUPPRESSOR OF CONSTANS 1 (SOC1). SOC1 activates floral meristem identity genes in the shoot apical meristem to initiate flower development. An accumulation of fully spliced and incompletely spliced *SOC1* transcript isoforms is observed in the *early flowering 9* (*elf9*) mutant that shows premature transition to flowering [110]. ELF9 codes for a homolog of human Tat stimulatory factor 1 (Tat-SF1) necessary for HIV replication and yeast CUS2, a splicing factor that aids the assembly of the splicing-competent U2 snRNP.

SUA (SUPPRESSOR OF *abi3-5*) is a splicing factor that affects seed maturation. The phytohormone ABA prevents germination, and during seed germination the ABA signaling component ABSCISIC ACID INSENSITIVE3 (ABI3) is down-regulated. ABI3 undergoes developmentally regulated alternative splicing that is under control of SUA, a homolog of the human splicing regulator RBM5 [111]. RBM5 interacts with U2AF^65^, and SUA has also been shown to interact with At-U2AF^65^, suggesting that it may act at an early step of the splicing process.

#### 2.3.3. The Nineteen Complex

A non-snRNP complex consisting solely of proteins is essential for the activation of the spliceosome, designated the nineteen complex (NTC) for its core component PRP19 [112]. Arabidopsis harbors two orthologs of PRP19 that interact with At-CDC5, the ortholog of the NTC components CELL DIVISION CYCLE 5 (CDC5) and PLEIOTROPIC REGULATORY LOCUS 1 (PRL1) [113]. Interestingly, several Arabidopsis orthologs of NTC components have been identified in genetic screens for components involved in pathogen defense. Plants sense the presence of pathogenic bacteria through dedicated receptors in the cell membrane, and the activation of appropriate signaling chains induces a set of defense responses in the plant. Transcriptional activation of defense genes largely relies on a key component, NONEXPRESSOR OF PATHOGENESIS RELATED GENES 1 (NPR1). Accordingly, *npr1* mutants are impaired in the induction of pathogenesis-related genes. A second site mutation of the *SNC1* (*SUPPRESSOR OF NPR1-1, CONSTITUTIVE1*) gene encoding a resistance (R) protein restores pathogen resistance in the *npr1* background [114]. In turn, a suppressor screen for mutants reverting the pathogen resistance phenotype of the *snc1* mutant revealed several mutants with a constitutive pathogen defense response even in the absence of a bacterial pathogen. Accordingly, the corresponding proteins are known as MOS (MODIFIER OF *snc1*) proteins. In this screen, At-BCAS2 was identified and designated MOS4. Moreover, through a proteomics approach to identified proteins associated with epitope-tagged MOS4 *in vivo*, the orthologs of PRP19 were recovered and designated MAC3A (MOS4 associated complex 3A) and MAC3B [113,115]. In the *mos4* and *cdc5* mutants and the *mac3a mac3b* double mutant the *SNC1* splicing pattern is altered, providing evidence that the MOS complex is involved in alternative splicing, similar to the NTC [116].

Recently, aberrant splicing of *SUPPRESSOR OF NPR1-1*, *CONSTITUTIVE4* (*SNC4*), and *CHITIN ELICITOR RECEPTOR KINASE 1* (*CERK1*), encoding receptor-like kinases involved in pathogen defense, has been found in the *sua* mutant [117]. Accordingly, *sua* mutants show enhanced susceptibility to *Pseudomonas syringae* infection. Thus, the RBM5 counterpart SUA appears to be involved both in germination (see Section 2.3.2) and response to pathogens.

Other proteins associated with the MOS (MODIFIER OF *snc1*) complex are MAC5A and MAC5B, with similarity to human RBM22 (RNA Binding Motif Protein 22) [118]. The zinc-finger protein RBM22 interacts with the U6 snRNA and the pre-mRNA in the spliceosomal B^act^ and C complexes [34,119]. This invites the speculation that MAC5 may also play a role in splicing in Arabidopsis.

Furthermore, the homolog of the human NTC component SNW/Ski-interacting protein domain protein (SKIP) and yeast Prp45 has been functionally characterized in Arabidopsis [120]. Arabidopsis At-SKIP interacts with the splicing factor SR45 and has a global effect on splicing. In particular, the core clock genes *PRR7* and *PRR9* are aberrantly spliced in *skip* mutants. This may contribute to the long period of the circadian clock in *skip* mutants. In addition, *skip* mutants are hypersensitive to both salt and osmotic stress [121]. A genome-wide analysis showed that At-SKIP impacts alternative splicing of numerous genes, particularly upon salt stress, and is required for splicing of genes associated with salt stress tolerance.

#### 2.3.4. The Nineteen Related Complex

The NTR (nineteen related complex) is another non-snRNP complex consisting solely of proteins. It contributes to spliceosome disassembly. Arabidopsis SPLICEOSOMAL TIMEKEEPER LOCUS1 (At-STIPL1) is a homolog of the NTR component TFP11 in humans and Ntr1p in yeast [122]. In the Arabidopsis *stipl1* mutant splicing of many introns is reduced. At the physiological level, *stipl1* shows a long period of the circadian clock. In accordance with this, the accumulation of circadian transcripts is altered. In particular, retention of intron 3 in the core clock gene *PRR9* is increased, which leads to a non-functional protein.

### 2.4. RNA Export Factors

In yeast and animals, the TREX (TRANSCRIPTION EXPORT) complex links transcription elongation to mRNA export. Human TREX has also been found to associate with the splicing machinery [123]. Recently, orthologs of components of the THO complex, a subunit of the TREX complex, have been connected to splicing in Arabidopsis. The *hrp1-5* mutant impaired in HYPER RECOMBINATION1 (HPR1)/THO1 is affected in bulk nuclear mRNA accumulation and thus RNA export [124]. In an allelic mutant, alternative splicing patterns of At-RS31, At-RS40, and At-SR34b are altered [125]. At-HRP1 co-localizes with At-SR33 in nuclear speckles. The exact mechanism of At-HRP1 function in splicing control remains to be determined. Mutants with reduced expression of Arabidopsis *At-THO2* also display splicing defects [126].

Mutants defective in the K homology (KH)-domain RNA-binding protein HOS5 (HIGH OSMOTIC STRESS GENE EXPRESSION 5) are impaired in mRNA export, particularly under salt stress conditions [127]. HOS5 interacts with At-RS40 and At-RS41 in nuclear speckles, and intron retention in many stress-related genes is observed under salt stress but not under normal conditions in the *hos5* mutant [127].

### 2.5. Miscellaneous Other RNA Binding Proteins

Arabidopsis RCF1 (REGULATOR OF C-REPEAT BINDING FACTOR GENE EXPRESSION 1) encodes a DEAD-box RNA helicase that is induced by cold [128]. Many cold-responsive genes are misspliced in the *rcf1-1* mutant under cold stress. Accordingly, the mutant is hypersensitive to cold stress.

The SR45a protein initially was considered to represent an SR protein, but now has been recognized to be a homolog of Transformer-2, a factor involved in sex-specific splicing of pre-mRNA from the sex determination gene doublesex in Drosophila [38,129]. SR45a interacts with several spliceosomal proteins, including U1-70K that recognizes the 5' splice site, U2AF^35^b that recognizes the 3' splice site, At-SR45, and At-SCL28, as well as the ortholog of PRP38 that contributes to maturation of the spliceosome before the first step of the splicing reaction. Thus, SR45a may participate in the splicing process in Arabidopsis through bridging the 5' end and 3' end of introns [130].

At-RTF2 encodes an evolutionarily conserved protein containing a Replication termination factor2 (Rtf2) domain [131]. While a homozygous null mutation in *At-RTF2* is embryo-lethal, RNA-seq performed on hypomorphic *atrtf2* alleles showed that 13%–16% of all introns are retained.

*ROOT INITIATION DEFECTIVE1* (*RID1*) encodes a DEAH-box RNA helicase similar to the splicing factors Prp22 in yeast and DEAH-box polypeptide8 (DHX8) in humans that is involved in remodeling of the spliceosome [132]. In the *rid1-1* mutant, several transcripts were aberrantly spliced and the mutant shows defects in leaf and root morphogenesis.

Mutants deficient in the subunits of the cap-binding complex (CBC), At-CBP20, and At-CBP80, which interact with the m^7^G cap of pre-mRNAs, also show widespread changes in splicing patterns [133,134]. In particular, splicing of the first intron is influenced in mutants deficient in the CBC.

## 3. Factors with a Dual Role in Splicing of Pre-mRNAs and microRNA Precursors

Several Arabidopsis splicing factors affect splicing of pre-mRNAs and biogenesis of microRNAs (miRNAs). miRNAs, 21–24 nucleotide-long single-stranded RNAs, are generated from RNA polymerase II transcripts with internal stem-loop structures, the pri (primary)-miRNAs that are processed in a step-wise manner by RNase III-like proteins to yield the mature miRNAs. Plant miRNA biogenesis differs from miRNA biogenesis in animals. While in animals miRNAs frequently localize to introns of pre-mRNAs or non-coding RNAs, most plant miRNAs are derived from independent transcription units. In Arabidopsis, both conversion of pri-miRNAs to stem-loop pre (precursor)-miRNAs and further processing to miRNA/miRNA* duplexes take place in the nuclear compartment and are executed by the same enzyme, DICER-LIKE1 (DCL1) [135]. Mature miRNAs are loaded into Argonaute (AGO)-containing RNA-induced silencing complexes and guide AGO proteins to complementary sites in their targets, leading to down-regulation of the transcripts predominantly by AGO cleavage in plants, but also by inhibition of translation [136].

Several RNA-binding proteins contribute to processing of the pri-miRNAs: the double-stranded RNA-binding protein HYPONASTIC LEAVES1 (HYL1), the zinc finger protein SERRATE (SE), a homolog of ARSENITE RESISTANT 2, and the G-patch domain protein TOUGH (TGH) [137]. Together with DCL1, these proteins form the so-called Microprocessor complex that is tethered to the pri-miRNA via interaction with the cap binding proteins At-CBP20 and At-CBP80 [134,138,139]. SE and HYL1 promote accurate pri-miRNA processing by DCL1 [140,141]. TGH enhances DCL1 activity without influencing the processing accuracy [137]. DAWDLE (DDL) is a forkhead-associated (FHA) domain protein that is assumed to recruit DCL1 to pri-miRNAs [142]. DDL is a homolog of SNIP1 (Smad nuclear interacting protein 1) that is recruited to the spliceosome at the transition from complex A to complex B in humans [143]. Up to now, DDL has not yet been associated with the splicing process in Arabidopsis.

The subunits of the CBC, At-CBP20, and At-CBP80 thus have a dual role in alternative splicing and pri-miRNA processing [133,134]. Similarly, SE is required for correct splicing of numerous pre-mRNAs [144]. There was a substantial overlap in the transcripts targeted by either SE or the CBC, and both SE and the nuclear CBC appeared to act in the selection of 5' splice sites and mainly on the first introns. Aberrant alternative splicing patterns were also found in the *hyl1-2* and *dcl1-7* mutants but these mutants shared little overlap with the *se* mutant. Thus, SE and CBC have distinct effects on alternative splicing from the other microRNA processing components DCL1 and HYL1. In addition to the general processing components, AtGRP7 also affects both pre-mRNAs and the processing of a subset of pri-miRNAs [145]. Furthermore, the NTC components CDC5 and PRL1 have been found to interact with the DCL-1 complex and enhance pri-miRNA processing [146,147]. Recently, STA1 has also been implicated in miRNA biogenesis, as intron-containing pri-miRNAs are incorrectly spliced in *sta1*, providing another example of a protein with dual function in pre-mRNA and pri-miRNA splicing [148].

## 4. Conclusions/Outlook

Relying on *Arabidopsis thaliana* as a model organism with ample genetic resources has greatly advanced our knowledge of plant splicing factors. In the absence of an *in vitro* splicing system, the analysis of mutants has proven revealing in elucidating a function of candidate RNA binding proteins in alternative splicing. Additionally, proteomic analysis of oligo(dT)-captured mRNPs has globally detected mRNA binding proteins in Arabidopsis [149]. However, mechanistic details on how individual factors work at the molecular level remain to be resolved. Furthermore, other splicing regulators may employ noncanonical RNA binding domains and thus may have been missed in computational predictions. For example, hundreds of proteins previously unknown to bind RNA have been identified in a proteomic approach in human cells [150]. Moreover, about half of the RRM-containing proteins in Arabidopsis appear to be specific to plants [151]. Thus, plants may have evolved their own specialized splicing regulators that are not recovered based on sequence similarity. Such additional regulators await experimental determination from genome-wide transcript profiling and target gene identification.
